# Competence of final year otolaryngology residents with the bedside head impulse test

**DOI:** 10.1186/s40463-019-0326-y

**Published:** 2019-01-18

**Authors:** D A Lelli, D Tse, J P Vaccani

**Affiliations:** 1 Department of Medicine, Division of Neurology, University of Ottawa, Ottawa, Canada; 2 Department of Otolaryngology - Head & Neck Surgery, University of Ottawa, Ottawa, Canada

**Keywords:** Head impulse test, Medical education, Competency based medical education, Neuro-otology

## Abstract

**Background:**

The bedside head impulse test (bHIT) is a clinical method of assessing the vestibulo-ocular reflex (VOR). It is a critical component of the bedside assessment of dizzy patients, and can help differentiate acute stroke from vestibular neuritis. However, there is evidence showing the bHIT is often not performed in appropriate clinical settings or is performed poorly. To date, there have been no studies evaluating the bHIT competence of graduating physicians.

**Methods:**

23 final year Otolaryngology –Head &Neck Surgery (OTL-HNS) residents in Canada were evaluated on the use of bHIT using a written multiple-choice examination, interpretation of bHIT videos, and performance of a bHIT. Ratings of subject bHIT performance were completed by two expert examiners (DT, DL) using the previously published Ottawa Clinic Assessment Tool (OCAT).

**Results:**

Using a cut-off of an OCAT score of 4 or greater, only 22% (rater DT) and 39% (rater DL) of residents were found able to perform the bHIT independently. Inter-rater reliability was fair (0.51, interclass correlation). The mean scores were 65% (14.1% standard deviation) on the video interpretation and 71% (20.2% standard deviation) on the multiple-choice questions. The scores on multiple choice examination did not correlate with bHIT ratings (Pearson r = 0.07) but there was fair correlation between video interpretation and bHIT ratings (Pearson r = 0.45).

**Conclusion:**

Final year OTL-HNS residents in Canada are not adequately trained in performing the bHIT, though low interrater reliability may limit the evaluation of this bedside skill. Multiple choice examinations do not reflect bHIT skill. These findings have implications for development of competency-based curricula and evaluations in Canada in critical physical exam skills.

**Electronic supplementary material:**

The online version of this article (10.1186/s40463-019-0326-y) contains supplementary material, which is available to authorized users.

## Background

The bedside head impulse test (bHIT) was described in 1988 as a quick and safe clinical method of assessing the angular vestibulo-ocular reflex (VOR) [1]. Because abnormalities of the VOR are a reliable sign of peripheral vestibular dysfunction, the bHIT has more recently been shown in multiple studies to be an essential part of the examination of patients presenting with both acute and chronic dizziness [1–4]. Despite this, the bHIT is often not performed in appropriate clinical settings, not done properly, or not interpreted appropriately [5]. Other studies have shown that the clinical usefulness of the bHIT improves with increasing experience at performing the test [6].

Competency based medical education represents a shift in medical education towards outcome based assessments to determine if competence has been achieved in specific clinical domains. Part of this process involves defining entrustable professional activities (EPAs), which are a broad unit of professional practice [7], which for OTL-HNS practitioners in Canada includes evaluating and managing a patient with dizziness [8]. Given the utility of the bHIT described above, performing and interpreting a bHIT forms a significant part of the competence of the training physician in evaluating dizzy patients.

To date, there have been no studies evaluating the competence of final year residents (postgraduate year 5) in performing the bHIT. Because performing a bHIT requires specialized examination skills often lacking in non-specialists [5], and because exposure to neuro-otology clinics is limited during residency, we hypothesize that most final year residents will not be competent in performing and / or interpreting the bHIT.

## Methods

All final year residents in Canadian OTL-HNS training programs were eligible for inclusion. Subjects were recruited and the study was performed at a final year review course given to Canadian OTL-HNS residents in Halifax, Canada. The only exclusion criterion was an active physical limitation that might restrict participants ability to perform the bHIT.

23 out of 31 eligible residents gave consent and participated in the study. Subjects were given a pre-test 10 item multiple-choice questionnaire (MCQ) to assess knowledge surrounding the clinical use of bHIT and how to perform the test (Additional file 1: Appendix A).

Subjects were asked to perform the bHIT on one of the authors (JPV), and instructed to perform the examination as they would during a typical clinical encounter. No feedback was given during the procedure, and competence in performing the procedure was judged by two expert evaluators watching the subjects perform the test in real time (DL, DT). The evaluators used a modified version of an entrustability scale, the OCAT, a previously validated tool for assessing clinical performance [9] based on a 5 point Likert scale. The bHIT was rated on multiple components: patient instructions, positioning, and impulse characteristics including speed, amplitude, consistency (between sides and with any repeated bHITs) and unpredictability (Additional file 1: Appendix B). One rating for each component was assigned for the entire encounter. Finally, the participants were shown a series of 10 bHITs on video monitors and asked to judge whether each bHIT was normal or abnormal.

A rating of 4 (“independent with only minor corrections”) was considered a reasonable cut-off for competent performance of the bHIT. Statistical analysis of the data included calculating percentages of residents in two scenarios: scoring 4 or greater on each aspect of the entrustability scale, or having a mean score of 4 or greater. Both were felt to be reasonable interpretations of being able to independently perform the procedure. Inter-rater reliabilities were calculated using inter-class correlations for both single rater reliability (how reliable a single rater’s scores would be) and the reliability of the mean rating of both raters (how reliable the mean of two raters scores would be). Mean scores were calculated for the MCQ and video interpretation tests, and Pearson correlations were calculated between the different forms of assessment: - mean rater bHIT scores, video interpretation score, and both mean MCQ scores and individual questions.

## Results

Only 5 (22%) and 9 (39%) of residents (DT and DL rating respectively) were able to perform the bHIT independently with an average score of 4 or greater. If a score of 4 or greater was required on each component of the bHIT, only 3 (13.6%) and 2 (9.1%) of residents would be judged as independent. Inter-rater reliability of the modified OCAT was fair (0.51, interclass correlation) and the reliability of the mean rating was good (0.67) for the continuous rating scale, though using a cutoff of an average of “4” or requiring “4” in each component for competency both resulted in poor inter-rater reliability (kappa = 0.19 and 0.33 respectively). Residents as a whole were rated poorest in proper use of distracting movements / unpredictability of the bHIT (mean score 3.20) and improper amplitude (mean score 3.24, mostly too large of an amplitude), though both means were less than one standard deviation from the overall mean bHIT score (3.64, standard deviation 0.64).

The mean scores were 65% (standard deviation 14.1%) for the video interpretation and 71% (20.2% standard deviation) for the multiple-choice questions. The scores on the multiple-choice examination did not correlate with mean bHIT rating scores (Pearson r = 0.07) but there was fair correlation between video interpretation and mean bHIT rating scores (Pearson r = 0.45). There was a single multiple choice question (regarding the definition of covert saccades, a component of physiology of a false negative bHIT) that had fair correlation (0.32) with bHIT ratings.

## Discussion

The results confirmed our hypothesis that the majority of final year Canadian OTL-HNS residents are not competent in performing the bHIT as judged by two expert examiners using the modified OCAT. Of the alternative assessment methods, only the subject’s ability to interpret the video recordings of bHIT correlated with judgment of clinical bHIT competency while multiple choice questions as a whole did not.

Interrater reliability was poor when making “competent or not” judgments based upon OCAT score cutoffs of “4”, and even treating the score as a continuous variable resulted only in fair (borderline poor) interrater reliability. This lack of agreement between raters raises concerns about evaluators’ ability to accurately assess competency for this bedside skill. The study design focused on rating different aspects of the bHIT as these were felt to be important in accurately assessing the skill. While this did provide information about which aspects of the skill were not performed well (amplitude, unpredictability), it likely contributed to the difficulty with judging competency as no measure of overall independence was recorded. Using a global rating of competence, doing rater training, and using more raters (using the reliability of the mean rating) are possible ways of improving reliability of competency judgments.

This study has several limitations. There were only two examiners from a single centre without formalized rater training, limiting judgments of true inter-rater reliability of the modified OCAT. Only OTL-HNS residents were evaluated in this study. OTL-HNS physicians are expected to be experts in the management of patients with dizziness. Other specialties also deal with dizzy patients (e.g: neurology and emergency medicine) and we may not be able to generalize to the competence of all residents treating dizziness with this study. This set of multiple choice questions showed poor validity as a substitute for observed clinical assessment, but it remains possible that a different set of questions may show better validity.

Despite these limitations, the findings have implications for the ongoing development of competency-based curricula in Canada. The poor correlation of competence with paper-based assessment of bHIT knowledge argues towards including clinic-based assessment of this skill as a specific component of an EPA addressing the evaluation of dizzy patients. Conversely, the interpretation of bHIT videos did show better correlation. Developing a more robust set of videos may allow improved rater training / consistency across multiple sites (i.e.: videos of subjects performing at various levels of competence) besides serving as an educational tool for trainees.

Future research directions could include studying residents in other subspecialties and including more examiners at different centres with different levels of expertise, with the goal to broaden the applicability of the findings. Development and validation of other assessment methods or rater training programs that improve interrater reliability are of paramount importance if decisions are to be made regarding competency. Finally, developing a teaching module could be integrated in both residency programs and continuing education in practice may help dissemination of this valuable clinical skill.

## Conclusions

Final year Canadian OTL-HNS residents are not competent in performing the bHIT, though poor interrater reliability may limit competence judgements. MCQs are not reliable in judging competence though interpretation of video bHITs may be. This has implications towards development of educational curricula for both residency programs and continuing education for physicians in practice. It clearly demonstrates the need for hands on assessments of learners and training of evaluators for more complex physical exam skills.

## Additional file

Additional file 1: **Appendix A.** Multiple Choice Questionnaire. **Appendix B.** Entrustment Scale. (DOCX 16 kb)

## Abbreviations

bHIT: Bedside head impulse test
EPA: Entrustable professional activity
MCQ: Multiple choice questionnaire
OCAT: Ottawa Clinic Assessment Tool
OTL-HNS: Otolaryngology – Head and Neck Surgery
