# Supplementary material for: Competence of final year otolaryngology residents with the bedside head impulse test
Source: J Otolaryngol Head Neck Surg. 2019 Jan 18;48:7. doi: 10.1186/s40463-019-0326-y (PMC6339336; doi:10.1186/s40463-019-0326-y)
Supplement: Supplementary file 1 — Appendix A. Multiple Choice Questionnaire. Appendix B. Entrustment Scale. (DOCX 16 kb) [file 40463_2019_326_MOESM1_ESM.docx]

# Appendix A – Multiple Choice Questionnaire

Please choose the BEST answer:

1) The bedside head impulse test (bHIT) is a measure of:

1. utricular function
2. angular vestibulo-ocular reflex
3. vestibulo-collic reflex
4. all of the above

2) The bHIT can be used to:

1. differentiate acute cerebellar stroke from vestibular neuritis
2. investigate presence of previous vestibular injury
3. assess acute and chronic dizziness patients
4. all of the above

3) The bHIT involves:

1. large amplitude head movements at low velocity
2. small amplitude head movements at low velocity
3. large amplitude head movements at high velocity
4. small amplitude head movements at high velocity

4) An abnormal right bHIT consists of:

1. a rightward head movement followed by a refixation saccade to the right
2. a rightward head movement followed by a refixation saccade to the left
3. a leftward head movement followed by a refixation saccade to the right
4. a leftward head movement followed by a refixation saccade to the left

5) A patient with significant bilateral vestibular hypofunction would likely show:

1. a bilateral abnormal bHIT
2. a bilateral normal bHIT as there is no net vestibular imbalance
3. an abnormal bHIT towards the side of the greater vestibular loss, and a normal bHIT on the other side
4. the bHIT is not interpretable in cases of bilateral vestibular hypofunction

6) In the presence of a right beating mixed horizontal-torsional jerk nystagmus, the most likely finding during head impulse testing would be:

1. an abnormal right bHIT
2. an abnormal left bHIT
3. a normal bHIT
4. an abnormal bHIT on both sides
5. the bHIT cannot be interpreted in the context of coexisting nystagmus

7) In the presence of upbeating jerk nystagmus, the most likely finding during head impulse testing would be:

1. an abnormal right bHIT
2. an abnormal left bHIT
3. a normal bHIT
4. an abnormal bHIT on both sides
5. the bHIT cannot be interpreted in the context of coexisting nystagmus

8) A covert saccade:

1. takes place while the eyelids are closed
2. is clinically detectable during bHIT
3. is a sign of uncompensated vestibular injury
4. happens during the head impulse movement

9) In the context of an acutely vertiginous patient, which of the following allows a near certain diagnosis of a vestibular neuritis?

1. An abnormal left and normal right bHIT, left beating nystagmus, and a skew deviation
2. A normal left and abnormal right bHIT, left beating nystagmus, and no skew deviation
3. A normal left and normal right bHIT, no nystagmus, and no skew deviation
4. A normal left and normal right bHIT, right beating nystagmus, and a skew deviation

10) Which of the following could make a bHIT uninterpretable?

1. Presence of a gaze-evoked nystagmus
2. A restriction of ocular motility
3. Ipsilateral posterior canal BPPV
4. All of the above

# Appendix B – Entrustment Scale

Scale

1: “ I had to do” i.e., Requires complete hands on guidance

2: “I had to talk them through” i.e., Able to perform task but requires constant direction

3: “I had to prompt them from time to time” i.e., Demonstrates some independence, but requires intermittent direction

4: “I needed to be in the room just in case” i.e., Independence but still requires supervision

5: “I did not need to be there” i.e., Complete independence, practice ready

1) Explanation/Instructions to the patient: 1 2 3 4 5

2) Overall body positioning/hand positioning: 1 2 3 4 5

3) Use of distracting movements / unpredictable thrusts: 1 2 3 4 5

4) Speed of head thrusts: 1 2 3 4 5

5) Amplitude of head thrusts: 1 2 3 4 5

6) Consistency of head thrusts: 1 2 3 4 5
